# Climate change effects on hydrologic processes and water quality in the Connecticut River watershed

**DOI:** 10.7717/peerj.21242

**Published:** 2026-06-16

**Authors:** Heather Parry, Timothy O. Randhir

**Affiliations:** Environmental Conservation, University of Massachusetts at Amherst, Amherst, MA, United States of America

**Keywords:** Watershed systems, Water quality, Hydrology, Swat, Modeling, Climate change

## Abstract

Increased frequency and intensity of extreme weather events, driven by climate change, are expected to alter hydrological and water quality processes in the Connecticut River watershed. This study aims to model the temporal and spatial impacts of climate change on the watershed’s hydrology and nutrient dynamics. We used the Hydrologic and Water Quality System (HAWQS), which incorporates the Soil and Water Assessment Tool (SWAT), to establish a baseline scenario and assess two climate scenarios: Coupled Model Intercomparison Project (CMIP5-) Representative Concentration Pathways (RCP) 4.5 and RCP 8.5. The model was validated using observed data from the United States Geological Survey (USGS) gage sites. Our results show that both climate scenarios will cause significant changes in hydrological processes, including a shift in precipitation seasonality, with more rainfall expected during winter and early spring. These changes will affect nutrient loading, shifting the seasonal peaks of nitrogen and phosphorus. Notably, the nitrogen-to-phosphorus ratio is projected to decrease across the entire watershed under both climate scenarios. These findings suggest that the altered hydrological regime and nutrient dynamics could have cascading effects on aquatic ecosystems, impacting phytoplankton and algal growth, with important implications for future nutrient management strategies.

## Introduction

The Intergovernmental Panel on Climate Change (IPCC) reports that the Earth has already warmed by 1.09 °C, with a greater than 50% chance of reaching 1.5 °C in the near future (2021–2040) (IPCC, 2022). The frequency and severity of extreme weather events, such as heavy rain and droughts, have been observed worldwide (IPCC, 2022). Prolonged droughts are worsening these extreme weather events (Burford et al., 2020). Most erosion occurs during significant, intense rainfall events (Edwards & Owens, 1991). Changes in rainfall patterns alter streamflow strength and timing, affecting freshwater systems (IPCC, 2022). Rising temperatures and changing rainfall patterns impact watershed systems (Ross & Randhir, 2022).

According to the United States Environmental Protection Agency (USEPA) National Water Quality Inventory Report, phosphorus and nitrogen are the main chemical stressors affecting rivers, lakes, and coastal waters in the United States (US) (US Environmental Protection Agency, 2017). The leading global sources of nitrogen and phosphorus include sewage, animal waste, atmospheric deposition, groundwater inflow, agricultural runoff, fertilizer runoff, and aquaculture (Anderson, Gilbert & Burkholder, 2002). In the United States, the primary sources of nutrients are stormwater runoff from urban areas, atmospheric deposition, agricultural activities, and wastewater (US Environmental Protection Agency, 2017).

Excessive nutrient loading presents a global threat to aquatic ecosystems. Surplus nutrients can disrupt aquatic biodiversity and biogeochemical processes. Reduced levels of dissolved oxygen, caused by high-biomass algal blooms, eutrophication, declines in sensitive species, and toxic algal blooms, can all be attributed to nutrient enrichment (Anderson, Gilbert & Burkholder, 2002; Woodward et al., 2012). The algal blooms caused by increased nutrient loads can lead to harmful algal blooms, which produce toxins that may result in poisoning or death in aquatic organisms and humans. Additionally, non-toxic blooms can cause other problems within ecosystems. Large biomass accumulation leads to low oxygen levels and shading of submerged vegetation, further contributing to eutrophication (Anderson, Gilbert & Burkholder, 2002).

Temperatures in the Northeastern United States have risen by 0.26 °C per decade since the 1970s, with winter temperatures increasing by more than 0.9 °C. These temperature rises are expected to continue, leading to more days of extreme heat and longer droughts (Resilient MA, 2018). Additionally, changes in the hydrological cycle are likely in the Northeastern US, with increased rainfall anticipated in spring and winter (USGCRP, 2017). This additional winter rainfall will reduce spring snowmelt and increase winter stormwater runoff. The reduction in snowmelt will also result in lower springtime flows. A slight decrease in summer precipitation, combined with rising temperatures, could cause more frequent droughts. The frequency of heavy rainfall events is predicted to increase, raising the risk of flooding (Resilient MA, 2018).

The main sources of nitrogen and phosphorus worldwide include sewage, animal waste, atmospheric deposition, groundwater inflow, agricultural runoff, fertilizer runoff, and aquaculture. These nutrient sources have caused a threefold increase in phosphorus transport to the oceans compared to pre-industrial times. Nitrogen levels have also risen significantly; for example, nitrogen levels have quadrupled in the Mississippi River and increased sixfold in the Chesapeake Bay over the past 40 years (Anderson, Gilbert & Burkholder, 2002). These increases have led to economic impacts, with the degradation of ecosystem services due to nutrient pollution and related stressors—such as eutrophication and oxygen depletion—costing the United States $2.2 billion annually (Dodds, 2007). In Massachusetts, blue–green algae blooms have become more frequent, with more than 30 bloom events by August 6 or 2025 in Massachusetts lakes and ponds (Mass.Gov., 2025). The increasing occurrence of blue–green algae blooms in recreational water bodies has raised concerns among public health officials and private citizens, as exposure to contaminated water can harm humans and animals (Jochem, 2019).

Climate change is likely to alter hydrological processes in the Connecticut River watershed significantly. These shifts in water quantity and precipitation timing are expected to impact water quality in both freshwater and marine environments. 

The goal of this study is to understand the temporal and spatial impacts of climate change on hydrology and nutrients in the Connecticut River watershed. Our research addresses this goal through three specific objectives: first, to establish baseline hydrological conditions using the SWAT model within the Hydrologic and Water Quality System (HAWQS) framework; second, to evaluate the effect of two climate change scenarios, RCP 4.5 and RCP 8.5, on hydrological and nutrient loading processes; and third, to analyze the dynamics of limiting nutrients at the sub-basin level. We hypothesize that projected climate change will cause significant shifts in seasonal precipitation patterns, leading to changes in streamflow and groundwater contributions. These shifts will, in turn, influence nutrient loading and the nitrogen-to-phosphorus ratio within the watershed, potentially impacting phytoplankton growth and eutrophication. This study demonstrates that these hydrological and nutrient changes will have cascading effects on the ecosystem, highlighting the importance of proactive nutrient management strategies.

## Methods

**Figure 1:**
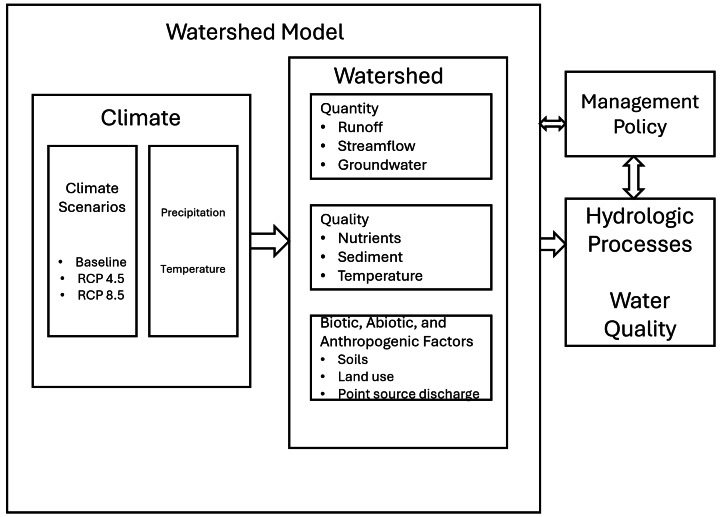
Conceptual model of this study.

The conceptual model depicting the information flows is presented in Fig. 1.

### Study area

**Figure 2:**
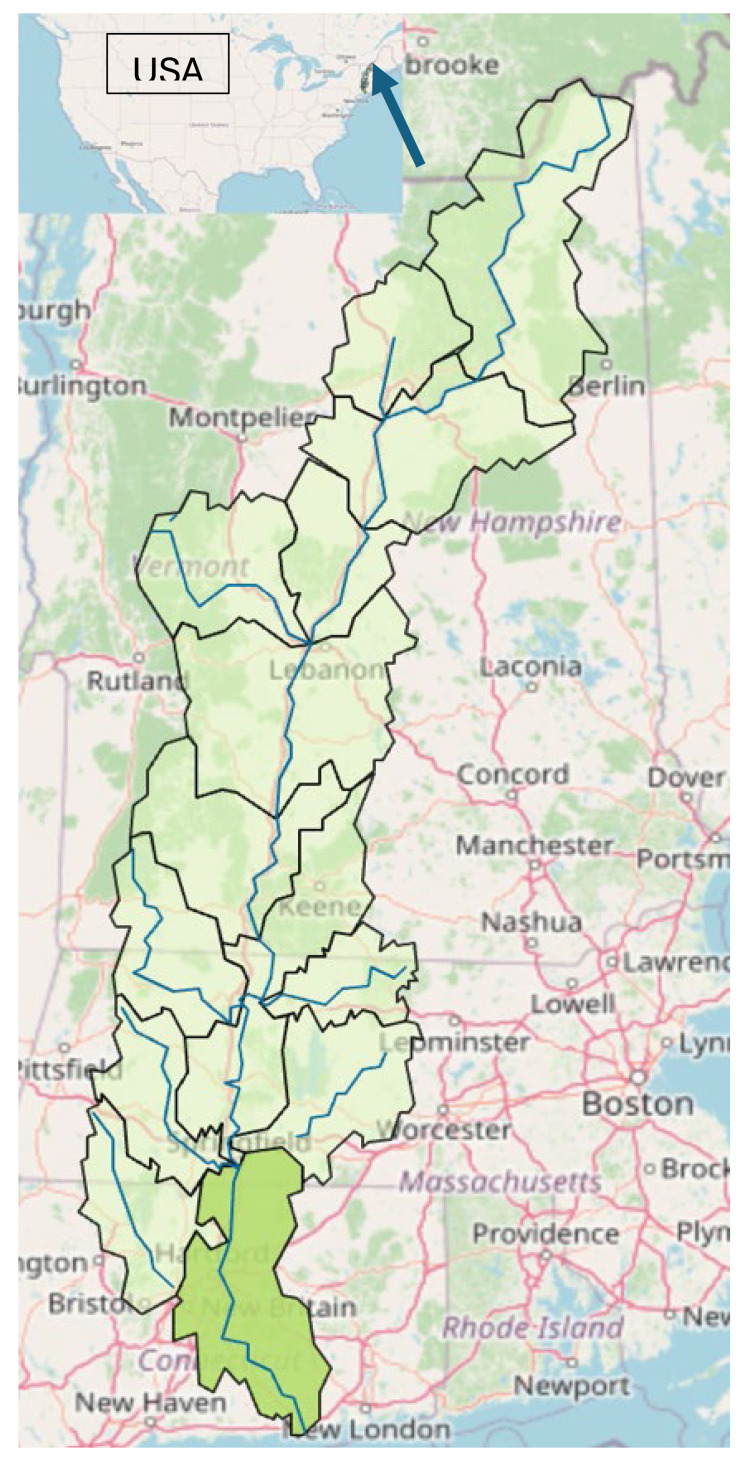
Connecticut River watershed.

The Connecticut River is the longest in New England, stretching about 410 miles (Fig. 2). It flows through four states in the region, starting at Fourth Connecticut Lake in northern New Hampshire and traveling south. It forms the border between New Hampshire and Vermont, crosses central Massachusetts, and ends in Old Lyme, Connecticut, where it flows into Long Island Sound. The Connecticut River supplies 70% of the freshwater that drains into Long Island Sound. Its watershed covers roughly 31,000 km^2^, mostly forested. The watershed land use is mixed with more forested areas in the northern portions of the watershed and transitions to more agricultural and urban uses towards south portions of the watershed (Paulding & Randhir, 2021). The average annual precipitation is 1,228.4 mm.

Blue–green algae, or cyanobacteria, have been observed in the Connecticut River and its tributaries (CT DEEP, 2020; Beskenis, 2006). These can become problematic when they grow large enough to form mats. Several invasive aquatic plants, such as Hydrilla, Eurasian milfoil, and water chestnut, are also found in the Connecticut River and its tributaries. About 15,000 years ago, Lake Hitchcock was formed as the ice sheet covering most of New England began to retreat. As the ice melted, the lake expanded and reached a length of 200 miles (Stone & Stone, 2012); its southernmost point was the sediment dam at Rocky Hill, CT. Large deltas of sand and gravel formed in the lake as rivers from the melting glacier fed into it. The fine sediments settled at the bottom. As Lake Hitchcock drained, the Connecticut River began to form by carving a channel into the sediments left behind by the lake. These sediments contribute to the fertile soil of the Connecticut River valley, making it highly suitable for farming. The valley has been farmed for over 1,000 years (CT River Conservancy). The river’s presence also attracted industries seeking to harness its power during the Industrial Revolution.

The Connecticut River has 16 major dams along the main stem; twelve of these are hydropower projects. There are also thousands of dams on the Connecticut River’s tributaries (CT River Conservancy).

### Hydrologic and climate modeling

For this study, the hydrology of the Connecticut River watershed was modeled using the widely used SWAT model (Zhao et al., 2021) within the operational framework of the Hydrologic and Water Quality System (HAWQS) (Yen et al., 2016). The SWAT model (Arnold et al., 2012) is used to simulate watershed processes in the baseline and two climatic scenarios. Water quantity results of interest include groundwater flow, surface flow, evapotranspiration, and streamflow, as these influence the nutrient cycle within the watershed (Marshall & Randhir, 2008). Water quality results affecting the nutrient cycle include nitrogen, phosphorus, sediment, and water temperature (Baker & Randhir, 2022; Marshall & Randhir, 2008). Both water quality and quantity impact the nitrogen-to-phosphorus ratio. This ratio, along with water temperature, acts as a trigger for algal blooms (Paerl & Huisman, 2009).

The SCS runoff equation (Soil Conservation Service, 1972) is used to estimate runoff under varying land-use and soil conditions (Rallison & Miller, 1981). The Modified Universal Soil Loss Equation (MUSLE) (Williams, 1975) is used to compute rainfall- and runoff-induced erosion (Neitsch et al., 2002). The Penman-Monteith Method (Monteith, 1965; Allen, 1986; Allen et al., 1989) was used to determine evapotranspiration. Groundwater simulation uses an equation developed by Hooghoudt (1940) that addresses the “steady-state response of groundwater flow to recharge” (Neitsch et al., 2002). SWAT uses the Bagnold equation (Bagnold, 1966) for sediment routing in a specific reach. SWAT models the nitrogen cycle in the soil profile and shallow aquifer and simulates the phosphorus cycle using six distinct phosphorus pools in the soil. The surface layer of soil has the highest nutrient content. Surface runoff can erode that layer, transporting nutrients from the surrounding land surface into the water bodies in the watershed. The processes modeled for nitrogen are comprehensive and include mineralization and decomposition/immobilization, nitrification and ammonia volatilization, denitrification, atmospheric deposition (wet and dry deposition), fixation, upward movement of nitrate in water in the first layer of soil, leaching, and nitrate in the shallow aquifer (Neitsch et al., 2002). SWAT uses organic and inorganic phosphorus pools to model the phosphorus cycle, including mineralization and decomposition/immobilization, humus mineralization, sorption of inorganic phosphorus, and leaching (Neitsch et al., 2002). Details on the assumptions and parameter estimation are presented in Arnold et al. (2012).

SWAT models the flow of nitrates within the watershed through surface, lateral, and percolation flows. Organic nitrogen is linked to sediment loading from the surrounding land as it adheres to sediment particles. The organic nitrogen transported to a water body with sediment is assessed using the loading function suggested by McElroy et al. (1976) and modified by Williams & Hann (1978). Due to the low mobility of soluble phosphorus in soil, SWAT models its movement from surface runoff in the top 10 mm of soil (Neitsch et al., 2002). Organic and mineral forms of phosphorus can attach to sediment, just as nitrogen does, which is carried with the sediment.

This study explores key hydrological and environmental variables, including streamflow, surface flow, groundwater flow, evapotranspiration, and sediment, nitrogen, and phosphorus levels. A baseline model was created using climate data from the National Oceanic and Atmospheric Administration (NOAA) and the National Weather Service (NWS). The model simulations ran from January 1, 1966, to December 31, 2018, with a five-year warm-up period to produce accurate daily SWAT outputs. Two climate scenarios were tested in the SWAT baseline model to assess the effects of climate change on water quantity and nutrient levels in the Connecticut River. The two emissions scenarios, RCP 4.5 and RCP 8.5, were simulated from 2022 to 2099 using the National Center for Atmospheric Research’s Community Climate System Model (CCSM) as the basis for this study, which employed CMIP5 Global Circulation Models (GCM) (Gent et al., 2011; Neale et al., 2013). CCSM-CMIP5 is subject to rigorous validation and integration into established downscaling frameworks, such as CORDEX (HAWQS, 2023).

Furthermore, it offers a more conservative, computationally accessible baseline and uses RCP pathways that are useful for connecting to existing policies. The selected GCMs include CanESM2 (from the Canadian Centre for Climate Modeling and Analysis), CCSM4 (Community Climate System Model version 4), GISS-E2-R (from the Goddard Institute for Space Studies), HadGEM2-ES (from the Met Office Hadley Centre), and MIROC5 (Model for Interdisciplinary Research on Climate) (Fant et al., 2017). The points of mean temperature and precipitation change across the Contiguous United States (CONUS) were plotted in the selection using scatter plots (*i.e.,* precipitation change on the *X*-axis and temperature on the *Y*-axis). The GCMs selected best represent the variability, or “scatter”, of the full set (Fant et al., 2017). The projections were downscaled using a statistical process that uses a multi-scale spatial matching scheme to select analog days from observations across CONUS (Fant et al., 2017).

The RCP 4.5 is considered the mid-level emission scenario and reflects greenhouse gas emissions incorporating mitigation strategies, projected to result in a radiative forcing change of 4.5 W/m^2^ by 2100. RCP 8.5 indicates a predicted radiative forcing increase of 8.5 W/m^2^ by 2100, representing the high-end emissions scenario without mitigation measures (HAWQS, 2020). We did not use RCP 2.5 to focus on average- and high-concentration pathways. Calibration and validation were compiled in HAWQS (Yen et al., 2016) as part of the operational framework development using streamflow observations from 65 gaging stations in the watershed, evaluated with Nash-Sutcliffe efficiency (NSE), percent bias (PBias), and the ratio of the root mean square error to the standard deviation of measured data (RSR) (Moriasi et al., 2007). The mean NSE was 0.61 (standard deviation, SD: 0.25), mean PBias was 8.99 (SD: 12.32), and mean KGE was 0.76 (SD: 0.12). The HUC 8 level includes 14 sub-basins within the Connecticut River watershed. Previous research in the watershed (Marshall & Randhir, 2008; Tsvetkova & Randhir, 2019) confirms the validation of the SWAT-HAWQS model used in this study.

## Results

**Figure 3:**
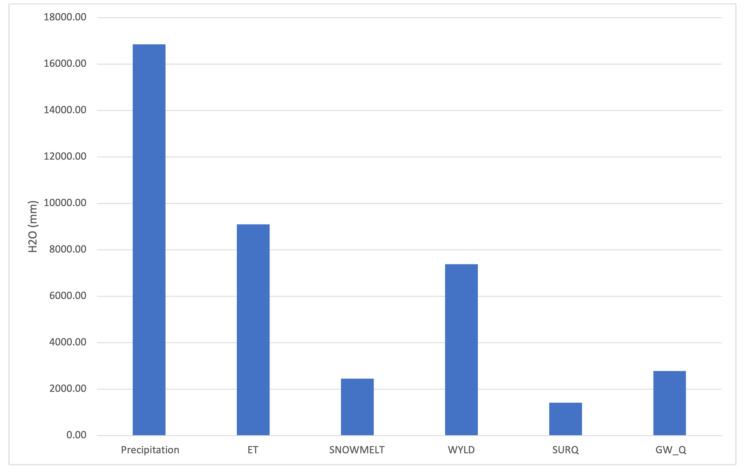
Annual water quantity variables in the baseline.

**Figure 4:**
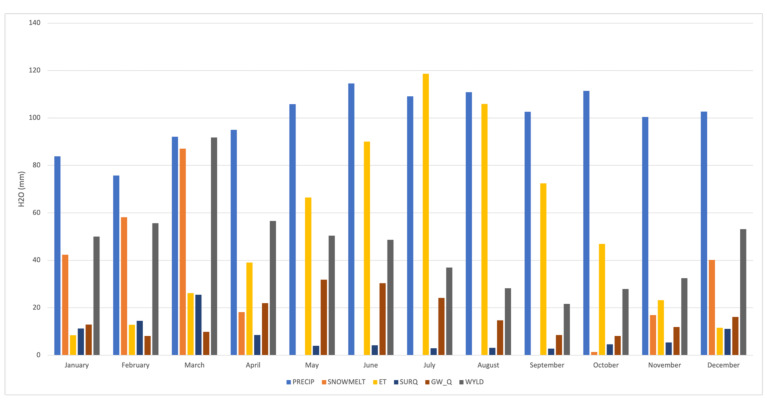
Monthly average water quantity variables in the baseline scenario.

The simulation results are presented as changes in water quantity and water quality in the study watershed. Water quantity variables include precipitation, snowmelt, evapotranspiration, surface flow, groundwater flow, and water yield. Water quality variables include sediment, total nitrogen, total phosphorus, dissolved oxygen, and chlorophyll a; their average annual values appear in Fig. 3. Parameters are: Precipitation—average total precipitation on subbasin (mm H2O); ET—actual evapotranspiration (mm H2O); SNOWMELT—snowmelt (mm); WYLD—net water yield to reach (mm); SURQ—surface runoff (mm); GW_Q—groundwater discharge to reach (mm). Monthly data show distinct seasonal patterns (Fig. 4, Table 1). Precipitation ranges from 75.7 mm in February to 114.5 mm in June. Snowmelt is 0 mm from June through September and peaks at 87.1 mm in March (monthly mean 22 mm). Evapotranspiration ranges from 8.5 mm in January to 118.7 mm in July (mean 51.8 mm month^−^^1^). Surface flow varies from 2.8 mm in September to 25.5 mm in March (mean 51.8 mm month^−^^1^). Groundwater flow is lowest in February and October (8.1 mm) and highest in May (16.5 mm). Water yield ranges from 21.7 mm in September to 91.8 mm in March (mean 46.1 mm month^−^^1^).

**Table 1:** Baseline monthly average water quantity.

	**Precipitation (mm H2O)**	**Snowmelt (mm H2O)**	**ET** **(mm H2O)**	**Surface runoff (mm H2O)**	**Groundwater discharge (mmH2O)**	**Water yield (mmH2O)**
January	83.8	42.4	8.5	11.3	12.9	50.0
February	75.7	58.1	12.8	14.5	8.1	55.6
March	92.1	87.1	26.2	25.5	9.8	91.8
April	95.0	18.2	39.1	8.5	21.9	56.6
May	105.9	0.1	66.5	4.0	31.8	50.4
June	114.5	0.0	90.0	4.2	30.3	48.7
July	109.1	0.0	118.7	2.9	24.2	36.9
August	110.9	0.0	105.9	3.1	14.7	28.2
September	102.6	0.0	72.4	2.8	8.6	21.7
October	111.4	1.4	47.0	4.6	8.1	27.9
November	100.4	16.9	23.2	5.4	11.9	32.5
December	102.7	40.2	11.6	11.1	16.2	53.2

**Figure 5:**
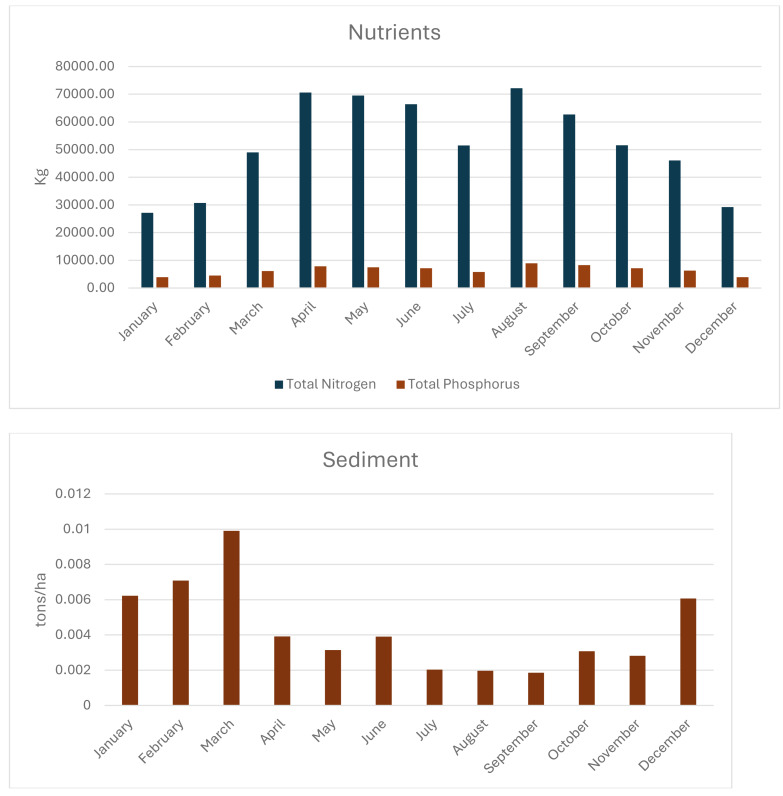
Monthly average water quality variables in the baseline scenario.

The average annual chlorophyll a is 192 kg, but is not shown in the figure. Monthly total nitrogen and total phosphorus (kg) are in Figs. 4 and 5, and Table 2. Total nitrogen and phosphorus reach minimum average monthly levels in January (27,142 kg and 3,935 kg), while sediment is lowest in December (8,129 metric tons). Sediment, total nitrogen, and total phosphorus in the baseline peak in August, and nutrient levels generally correlate positively with sediment and erosion (McElroy et al., 1976).

### Climate change scenarios

**Figure 6:**
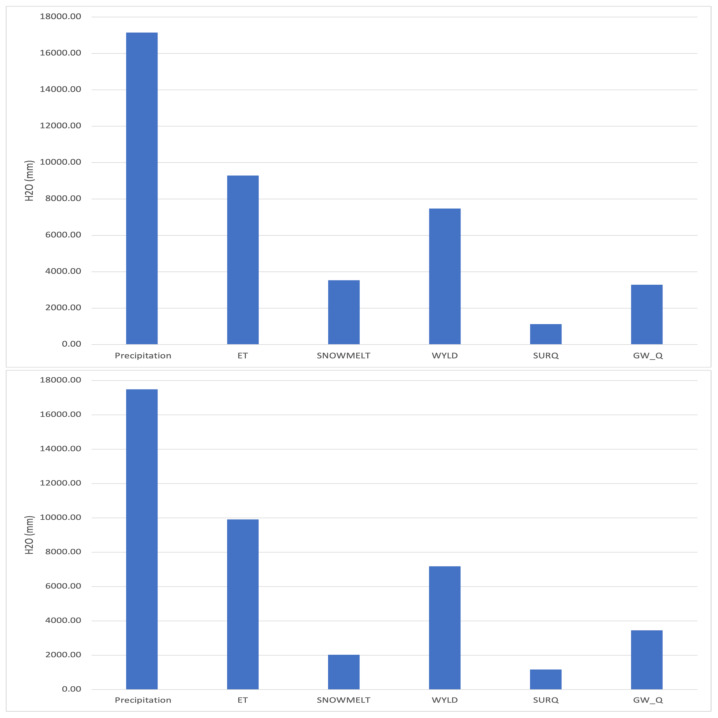
Annual water quantity variables for the climate scenarios RCP 4.5 and 8.5.

The annual water quantity under two climate scenarios is shown in Fig. 6. Yearly subbasin averages for each variable are compared with the baseline. Under RCP 4.5, annual averages increase for precipitation (2%), evapotranspiration (2%), snowmelt (44%), and water yield (1%), and decrease for surface flow (−21%) and sediment exported (−68%). Under RCP 8.5, precipitation and evapotranspiration increase (4% and 9%), while snowmelt (−17%), water yield (−3%), surface runoff (−17%), and sediment exported (−2%) all decline relative to baseline.

**Figure 7:**
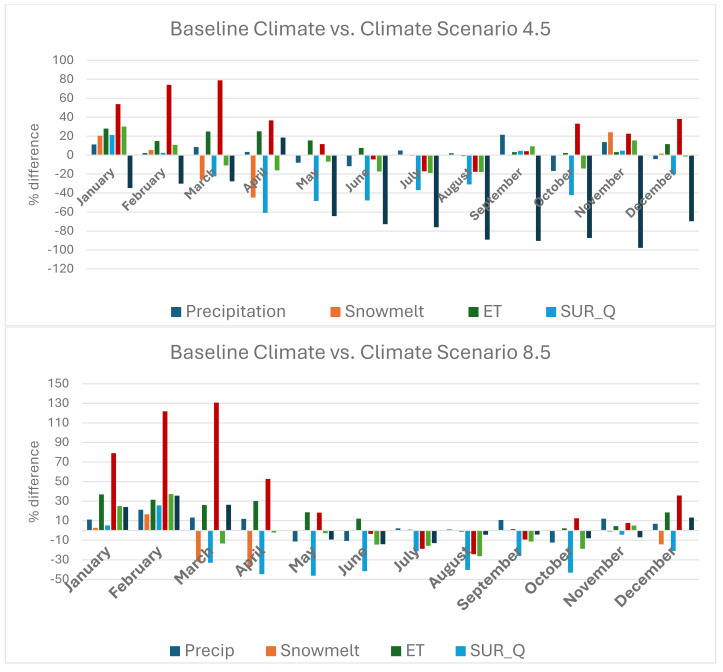
Monthly average percent difference of water quantity variables from the baseline.

**Figure 8:**
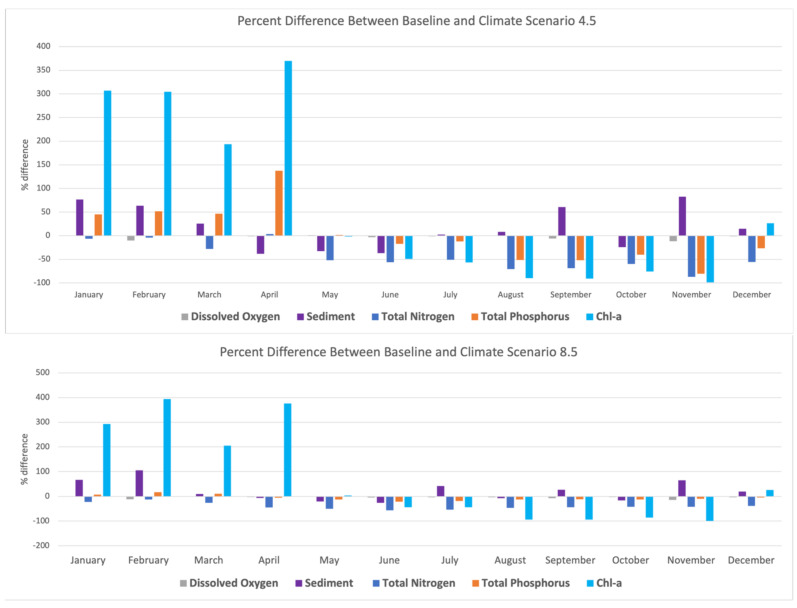
Monthly average percent difference of water quality variables from the baseline.

Monthly behavior under RCP 4.5 and RCP 8.5 (Tables 3 and 4; Figs. 7 and 8) broadly resembles the baseline, with shifts in timing and magnitude. In RCP 4.5, precipitation is highest in September (124 mm) and lowest in February (77.4 mm); snowmelt peaks in March (64.7 mm) and is zero from June to September. Evapotranspiration peaks in July (117.9 mm) and is lowest in January (10.8 mm). Surface runoff is highest in March (19.8 mm) and lowest in July (1.9 mm). Groundwater discharge is highest in May (35.5 mm) and lowest in September (8.9 mm). Water yield peaks in March (81.9 mm) and is lowest in August (23.2 mm). Under RCP 8.5, maximum precipitation occurs in September (113.7 mm) and minimum in February (91.8 mm). Snowmelt is zero from June to September and peaks in February (67.8 mm). Evapotranspiration is highest in July (119.8 mm) and lowest in January (11.9 mm). Surface runoff peaks in February (18.2 mm); surface runoff, groundwater discharge, and water yield are all lowest in September (2.1 mm, 7.8 mm, and 19.2 mm). Groundwater discharge is highest in May (37.6 mm), and water yield peaks in March (79.6 mm). The shift in snowmelt seasonality under climate change agrees with earlier work and has implications for streamflow timing and winter nutrient leaching from shortened snowpack seasons (Marshall & Randhir, 2008; Brooks et al., 2011; Grimm et al., 2013a; Grimm et al., 2013b).

**Table 2:** Baseline average monthly water quality.

	**Sediment** ** (metric tons)**	**Flow** ** (ms3/s)**	**Chl-a** ** (kg)**	**Nitrogen** ** (kg)**	**Phosphorus** ** (kg)**	**Nitrogen Conc.** ** (mg/L)**	**Phosphorus Conc.** **(mg/L)**	**DisOX Conc.** ** (mg/L)**
January	8,552	250	60	27,142	3,935	1.50	0.22	13.92
February	10,095	287	88	30,721	4,500	1.38	0.20	13.77
March	16,419	439	215	48,969	6,150	1.33	0.17	13.19
April	21,429	604	370	70,556	7,863	1.35	0.15	11.86
May	20,352	598	308	69,555	7,465	1.33	0.15	10.43
June	18,480	571	237	66,393	7,122	1.33	0.15	9.52
July	15,393	462	119	51,446	5,770	1.29	0.15	9.22
August	23,884	625	299	72,139	8,873	1.34	0.17	9.35
September	20,635	551	239	62,651	8,287	1.33	0.18	10.04
October	17,447	452	165	51,517	7,117	1.35	0.19	11.19
November	15,327	393	147	46,060	6,266	1.39	0.19	12.57
December	8,129	264	63	29,249	3,950	1.45	0.20	13.63

In RCP 4.5, water quality variables peak in spring: sediment, total nitrogen, and total phosphorus reach their highest monthly values in April (25,394 metric tons, 73,059 kg, and 18,655 kg). Minimum sediment, nitrogen, and phosphorus occur in November (339 metric tons, 5,942 kg, and 1,216 kg). The lowest values for sediment, nitrogen, and phosphorus overall occur in December (9,208 metric tons, 18,006 kg, 3,756 kg). Sediment peaks in August (22,872 metric tons); nitrogen peaks in April (39,254 kg); and phosphorus peaks in August (7,716 kg).

Monthly percentage differences between climate scenarios and baseline (Fig. 7) highlight seasonal shifts. Annual precipitation exceeds baseline under both scenarios, increasing by 2% in RCP 4.5 and 4% in RCP 8.5. In RCP 4.5, precipitation generally rises from January to April, decreases in May–June, then fluctuates with strong increases in September (21%) and November (14%). In RCP 8.5, precipitation increases more consistently from November to April (12%, 7%, 11%, 21%, 13%, 12%), declines in May–June (−11% each), and shows smaller increases in July–August (2% and 1%). Annually, snowmelt increases by 44% in RCP 4.5 but decreases by 17% in RCP 8.5, with more snowmelt shifting into January–February and reductions later in the snowmelt season.

Evapotranspiration increases each year in both scenarios relative to baseline (2% in RCP 4.5, 9% in RCP 8.5), with the largest gains in winter and early spring (December–May), especially in December (28% and 37% in RCP 4.5 and RCP 8.5, respectively). These patterns are consistent with prior Connecticut River watershed studies and broader climate projections linking higher temperatures to increased evapotranspiration.

Annual surface flow decreases in both climate scenarios, aligning with earlier work on the basin. In January and February, surface flow exceeds baseline (RCP 4.5: +21% and +2%; RCP 8.5: +5% and +26%), but it declines in most other months, except for modest increases in September (4%) and November (5%) under RCP 4.5. Groundwater contributions to streamflow increase annually under both scenarios (RCP 4.5: +18%; RCP 8.5: +5%), with the largest increases in winter–spring and especially in March (79% and 130%). Water yield rises annually by 1.3% in RCP 4.5 and falls by 3% in RCP 8.5, with most increases concentrated in winter (notably November, January, and February) and declines during the rest of the year, especially midsummer (July–August).

Annual dissolved oxygen declines by about 4% under both climate scenarios, with monthly values generally lower than baseline except for a slight May increase in RCP 4.5 (Tables 5 and 6). Average annual sediment export decreases markedly in RCP 4.5 (−68%) and slightly in RCP 8.5 (−2%). In both scenarios, sediment increases in winter and early spring (large positive anomalies from November to March), then decreases in April–June, and often increases again in late summer (July–September).

**Table 3:** Climate change scenario RCP 4.5 monthly average water quantity.

	**Precipitation (mm H2O)**	**Snowmelt (mm H2O)**	**ET** **(mm H2O)**	**Surface runoff** **(mm H2O)**	**Groundwater discharge (mm H2O)**	**Water yield** **(mm H2O)**
January	93.1	51.0	10.8	13.7	19.9	65.0
February	77.4	61.3	14.7	14.9	14.1	61.7
March	100.0	64.7	32.7	19.8	17.6	81.9
April	98.2	10.0	48.9	3.3	29.9	47.5
May	97.4	0.6	76.7	2.1	35.5	46.9
June	101.2	0.0	96.8	2.2	28.9	40.3
July	114.4	0.0	117.9	1.9	20.0	30.0
August	113.1	0.0	104.6	2.1	12.2	23.2
September	124.5	0.0	74.7	2.9	8.9	23.7
October	93.0	2.8	47.9	2.6	10.8	24.0
November	114.1	21.0	23.9	5.6	14.5	37.5
December	98.4	40.8	12.9	8.9	22.3	52.4

**Table 4:** Climate change scenario 8.5 monthly average water quality.

	**Precipitation (mm H2O)**	**Snowmelt (mm H2O)**	**ET** **(mm H2O)**	**Surface runoff (mm H2O)**	**Groundwater discharge (mmH2O)**	**Water yield (mmH2O)**
January	93.1	43.5	11.6	11.9	23.1	62.5
February	91.8	67.8	16.9	18.2	18.0	76.3
March	104.2	59.2	33.0	17.1	22.7	79.6
April	106.4	11.6	50.8	4.7	33.5	55.4
May	94.0	0.3	78.8	2.2	37.6	49.0
June	102.4	0.0	100.9	2.5	29.3	41.7
July	111.6	0.0	119.8	2.3	19.6	31.1
August	112.1	0.0	104.6	1.9	11.2	20.8
September	113.7	0.0	73.5	2.1	7.8	19.2
October	97.7	3.5	48.0	2.6	9.1	22.7
November	112.6	16.7	24.2	5.1	12.8	34.1
December	109.8	34.4	13.7	8.8	22.0	53.1

**Table 5:** Climate change scenario RCP 4.5 monthly average water quality.

	**Sediment (metric tons)**	**Flow (ms3/s)**	**Chl-a (kg)**	**Nitrogen (kg)**	**Phosphorus (kg)**	**Nitrogen Conc. (mg/L)**	**Phosphorus Conc. (mg/L)**	**DisOX Conc. (mg/L)**
January	5,578	448	246	25,351	5,705	0.81	0.17	13.91
February	7,049	545	354	29,434	6,828	0.68	0.15	12.36
March	11,902	795	633	35,270	9,001	0.57	0.14	13.22
April	25,394	1,356	1,740	73,059	18,655	0.64	0.16	11.71
May	7,259	582	303	33,533	7,574	0.70	0.16	10.52
June	5,015	449	121	29,094	5,907	0.78	0.16	9.25
July	3,681	359	52	25,317	5,069	0.89	0.18	9.08
August	2,597	275	31	21,222	4,304	1.03	0.20	9.27
September	1,979	223	23	19,502	3,977	1.18	0.23	9.44
October	2,197	239	40	20,598	4,267	1.26	0.25	11.10
November	339	55	2	5,942	1,216	1.89	0.36	11.14
December	2,456	232	80	13,049	2,907	1.10	0.22	13.45

**Table 6:** Climate change scenario RCP 8.5 monthly average water quality.

	**Sediment (metric tons)**	**Flow (ms3/s)**	**Chl-a (kg)**	**Nitrogen (kg)**	**Phosphorus (kg)**	**Nitrogen Conc. (mg/L)**	**Phosphorus Conc. (mg/L)**	**DisOX Conc. (mg/L)**	
January	10,605	439	237	21,064	4,217	1.01	0.19	13.65	
February	13,673	629	432	26,726	5,241	0.93	0.18	12.14	
March	20,715	832	658	36,239	6,794	0.83	0.15	13.12	
April	21,513	1,411	1,763	39,254	7,412	0.77	0.14	11.49	
May	18,496	613	319	34,494	6,491	0.73	0.14	10.34	
June	15,880	467	134	28,868	5,595	0.70	0.13	9.08	
July	13,396	376	67	23,900	4,678	0.72	0.14	8.91	
August	22,872	261	18	38,429	7,716	0.82	0.16	9.02	
September	19,798	199	14	34,955	7,319	0.84	0.17	9.30	
October	16,060	211	23	29,661	6,206	0.90	0.19	10.86	
November	14,255	47	1	26,790	5,656	0.93	0.19	10.81	
December	9,208	214	79	18,006	3,756	0.95	0.19	13.14	

Total nitrogen decreases annually under both scenarios (−47% in RCP 4.5, −43% in RCP 8.5), with monthly declines except for a 4% April increase in RCP 4.5. Total phosphorus also decreases on an annual basis (−3% in RCP 4.5, −8% in RCP 8.5). Monthly RCP 4.5 results show phosphorus increases from January to May (45%, 52%, 46%, 137%, and 1%), while RCP 8.5 shows phosphorus increases only in winter (January–March: 7%, 16%, 10%) and declines thereafter. Chlorophyll a decreases annually in both scenarios, with monthly values showing winter–early spring increases (January–April).

Precipitation and temperature strongly shape the hydrologic cycle, and projected precipitation totals increase in both RCP 4.5 and RCP 8.5 relative to baseline, especially in winter and early spring, altering snowmelt. Higher winter temperatures and winter rainfall tend to increase winter runoff, consistent with broader regional and global assessments. In this study, winter surface runoff increases with precipitation and snowmelt, and peak snowmelt and runoff shift from March in the baseline and RCP 4.5 to February in RCP 8.5, indicating a climate-driven change in the hydrologic regime. Evapotranspiration increases under both scenarios, likely reflecting higher temperatures, and may help explain the counterintuitive decrease in annual surface flow despite higher precipitation. These changes in precipitation timing and intensity are expected to alter hydrologic regimes and associated water quality (IPCC, 2022; USGCRP, 2017). In this study, key water quality indicators (sediment, nitrogen, phosphorus, chlorophyll a, dissolved oxygen) generally decline, even though higher precipitation typically enhances surface runoff and pollutant transport.

Increased evapotranspiration can diminish surface flow and modify seasonality. Nitrogen peaks in August in the baseline, shifts to April under RCP 4.5, and remains spring-dominated under RCP 8.5, while phosphorus still peaks in August and sediment peaks in August across all three climate scenarios. Sediment strongly influences nutrient delivery, particularly for phosphorus, which is typically sediment-bound; however, this study shows reduced phosphorus loads despite sediment increases in some months, suggesting altered source–transport relationships (Howarth et al., 2006; Sharpley & Syers, 1979; Neitsch et al., 2002). Under RCP 8.5, nitrogen decouples from sediment, consistent with its greater mobility and weaker sediment association. Shifts in nutrient ratios can favor different species and alter community composition, as documented in previous work.

**Figure 9:**
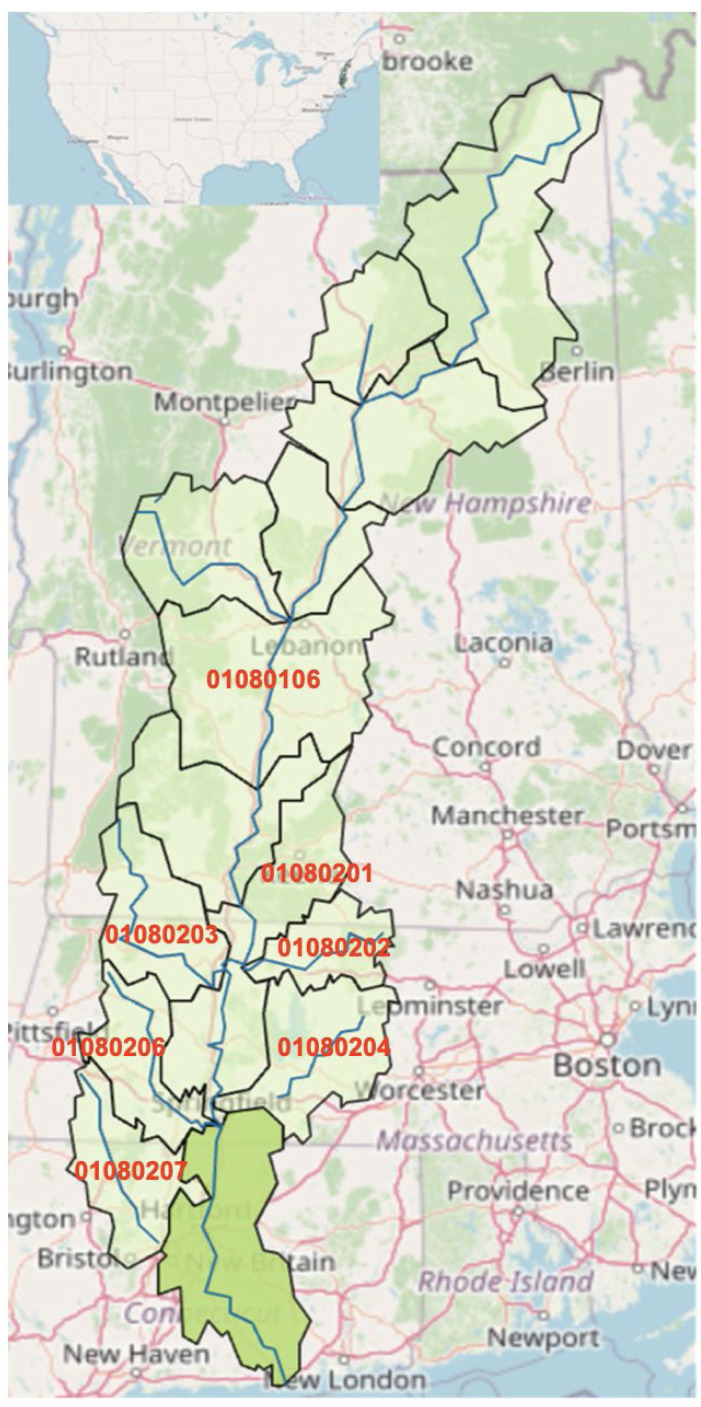
Connecticut River watershed with HUC 9 subwatershed delineations.

Nitrogen-to-phosphorus ratios were computed for all 14 HUC-8 subbasins in the Connecticut River watershed (Table 7). Summary statistics were derived for each subbasin and all three climate scenarios. Under both climate scenarios, all subbasins show lower N/P ratios than baseline, and both the range and maximum N/P values decline. Subbasins 01080106, 01080201, 01080202, 01080203, 01080204, 01080206, and 01080207 have reduced N/P ratios under both scenarios, but exhibit higher ranges and maxima under RCP 8.5 than under RCP 4.5 (Fig. 9).

**Table 7:** N/P ratios for the connecticut river watershed under baseline, RCP 4.5, and RCP 8.5 climate scenarios.

**N/P ratios**	** Baseline**	**RCP 4.5**	**RCP 8.5**
Mean	8.01	4.75	5.11
Standard Error	0.01	0.004	0.004
Median	7.80	4.80	5.02
Mode	6.67	0	5
Standard Deviation	1.57	0.79	0.61
Sample Variance	2.45	0.63	0.37
Kurtosis	0.87	8.68	2.86
Skewness	0.72	−1.56	0.94
Range	16.51	9.64	6.63
Minimum	0	0	2.62
Maximum	16.51	9.64	9.25

## Discussion

This study shows that climate change is likely to restructure the hydrologic regime and water-quality dynamics of the Connecticut River watershed, with stronger winter–spring hydrologic activity, reduced growing-season flows, and substantial shifts in nutrient timing and stoichiometry. Baseline simulations depict a snowmelt-dominated system, with peak precipitation, snowmelt, surface runoff, and water yield in late winter and early spring, and reduced flows and water yield in late summer and early fall—patterns consistent with other snow-influenced watersheds in the northeastern United States (Marshall & Randhir, 2008; Talib & Randhir, 2017). Under both RCP 4.5 and RCP 8.5 scenarios, annual precipitation and evapotranspiration increase, but surface runoff and, under RCP 8.5, water yield decline relative to the baseline, indicating that a larger share of precipitation is partitioned into evapotranspiration and subsurface storage rather than into direct overland flow. This combination of higher precipitation, higher evapotranspiration, and reduced runoff has been reported in other climate–watershed modeling studies and is consistent with projections of intensified water-cycle processes under warming (IPCC, 2022; USGCRP, 2017; US Geological Survey (USGS), 2025).

A key hydrologic signal is the timing of snowmelt and peak runoff. In the baseline and RCP 4.5 scenarios, peak snowmelt and surface runoff occur in March, whereas under RCP 8.5, they occur in February, reflecting warmer winters and more rainfall on snow. Similar shifts toward earlier snowmelt and peak flows have been documented in temperate and boreal basins worldwide and are expected to increase winter flood risk while reducing late-spring and summer base flows (IPCC, 2022; Grimm et al., 2013a; Grimm et al., 2013b). In this study, both RCP scenarios show enhanced winter and early-spring groundwater discharge, with the largest percentage increases in March, suggesting that recharge processes are also being pulled earlier into the hydrologic year. These results align with regional assessments projecting increases in cold-season precipitation and extreme rainfall, with associated increases in infiltration and groundwater recharge in parts of the Northeast (USGCRP, 2017; US Geological Survey (USGS), 2025).

Although annual precipitation increases under both scenarios, annual surface runoff declines relative to the baseline, especially under RCP 4.5. Such behavior contradicts the intuitive expectation that “more rain means more runoff.” However, it is consistent with mechanistic understanding that higher temperatures and longer growing seasons increase evapotranspiration and can deepen soil moisture deficits, thereby reducing the proportion of precipitation that becomes quick flow (Marshall & Randhir, 2008; Talib & Randhir, 2017). Model results from other regions likewise show that warming can offset or reverse the runoff effects of modest increases in precipitation, especially where soils are thick, vegetation is extensive, and subsurface pathways dominate (IPCC, 2022; USGCRP, 2017). In our simulations, water yield increases modestly under RCP 4.5 but declines under RCP 8.5, highlighting the basin’s nonlinear sensitivity to climate forcing. In both scenarios, however, water yield increases in winter and decreases across most of the growing season, so flow availability is shifted away from periods of highest ecological and human demand—a pattern consistent with projections of enhanced intra-annual variability in North American basins (USGCRP, 2017; US Geological Survey (USGS), 2025).

These hydrologic regime shifts have important implications for water quality. The model indicates substantial reductions in annual sediment export under both scenarios, especially under RCP 4.5, despite pronounced winter and early-spring peaks in sediment transport. This combination—fewer but more intense sediment export events—resembles findings from other watersheds where extreme storms drive large but episodic erosion and particulate transport, while changes in land cover, channel stability, and antecedent soil conditions limit annual loads (Sharpley et al., 2013; Gassman et al., 2007). In our case, enhanced winter flows and snowmelt likely mobilize sediment during a relatively short period, whereas lower summer runoff and increased infiltration reduce sediment delivery during the rest of the year.

Nutrient dynamics follow similarly complex patterns. Both climate scenarios predict sizable decreases in annual total nitrogen and total phosphorus relative to the baseline, while chlorophyll a and dissolved oxygen also decline. These declines in annual nutrient loads and algal biomass contrast with many eutrophication case studies where increased precipitation and runoff tend to amplify nutrient export and algal growth (USGCRP, 2017; Howarth et al., 2011). Several interacting mechanisms may explain the modeled response. First, the shift from surface runoff to greater evapotranspiration and subsurface flow pathways can reduce the transport of sediment-bound phosphorus, as surface runoff is often the dominant carrier of particulate P from uplands to streams (Sharpley & Syers, 1979; Haygarth & Jarvis, 2000). Second, lower summer flows combined with longer residence times may enhance in-stream uptake, denitrification, and sedimentation, thereby reducing downstream nutrient export (Grimm et al., 2013a; Grimm et al., 2013b; Howarth et al., 2011). Third, regional and global syntheses suggest that climate-induced changes in plant growth, soil organic matter turnover, and microbial processes can alter nutrient supply and retention, sometimes counteracting simple load–runoff relationships (IPCC, 2022; Grimm et al., 2013a; Grimm et al., 2013b).

The modeled seasonal nutrient behavior further underscores the importance of hydrologic timing. In the baseline scenario, nitrogen levels peak in late summer, whereas under both RCP scenarios, they peak in spring. In contrast, phosphorus retains a late-summer maximum across all scenarios. This decoupling suggests that nitrogen becomes more strongly coupled to snowmelt-driven flows and spring mineralization. At the same time, phosphorus remains linked to warm-season processes such as sediment resuspension, soil P mobilization during convective storms, or internal loading from sediments (Sharpley et al., 2013; Bruland & Richardson, 2006). As a result, nitrogen-to-phosphorus (N:P) ratios decline across all subbasins under both climate scenarios, with narrower ranges and lower maxima than in the baseline.

Shifts in N:P ratios are ecologically significant because phytoplankton, periphyton, and macrophyte communities respond strongly to nutrient stoichiometry, with different taxa and bloom types favored under different ratios (Howarth et al., 2011; Burkholder et al., 2001). Laboratory and field studies indicate that imbalances in N:P ratios and changes in nutrient forms (*e.g.*, dissolved *vs.* particulate, inorganic *vs.* organic) can foster shifts toward nuisance or harmful algal blooms, even when total nutrient loads remain constant (Burkholder et al., 2001; Burkholder & Glasgow, 1997). In this watershed, declining N:P ratios, coupled with reduced chlorophyll a, suggest a tendency toward lower overall algal biomass but potentially altered species composition and the timing of productivity pulses. The simultaneous decline in dissolved oxygen, however, indicates that climate-driven thermal and hydrologic changes (warmer water, reduced summer baseflows, longer residence times) may offset the benefits of lower nutrient loads by enhancing metabolic demand and reducing oxygen solubility (Grimm et al., 2013a; Grimm et al., 2013b; Howarth et al., 2011).

Collectively, the modeled changes in hydrologic regime and water quality align with broader assessments that climate change will intensify seasonality, shift flow timing, and complicate the relationship between nutrient loading and ecological response (IPCC, 2022; USGCRP, 2017; US Geological Survey (USGS), 2025). For the Connecticut River, increased winter and early-spring flows, earlier snowmelt, and reduced summer water yield are consistent with prior analyses that found greater cold-season runoff and higher summer low-flow risk under future climates (Marshall & Randhir, 2008; Talib & Randhir, 2017). At the same time, reductions in annual nitrogen, phosphorus, and sediment loads contrast with some regional scenarios that project increased runoff and nutrient export under more intense precipitation regimes, underscoring the importance of watershed-specific characteristics and the representation of subsurface processes in models (Talib & Randhir, 2017; IPCC, 2022).

These findings carry several management implications. First, the shift toward a more “flashy” winter–spring regime and diminished summer flows suggests that traditional design assumptions for flood control, reservoir rule curves, and environmental flow targets may become increasingly outdated. Regional assessments already recommend revisiting storage and release strategies to buffer higher winter inflows while sustaining ecological flows through longer, drier summers (IPCC, 2022; USGCRP, 2017; US Geological Survey (USGS), 2025). For the Connecticut River, this may involve increasing winter drawdown capacity, prioritizing groundwater recharge and headwater wetland conservation, and integrating climate-informed flow thresholds into operational rules.

Second, the decoupling between precipitation and nutrient export implies that water-quality management cannot rely solely on annual load reductions. Although modeled annual loads of nitrogen, phosphorus, and sediment decline, more episodic cold-season pulses and shifts in N:P ratios can still restructure aquatic communities and oxygen dynamics, particularly in impounded or low-gradient reaches where residence times are long and internal loading is significant (Sharpley et al., 2013; Howarth et al., 2011). Adaptive strategies should therefore emphasize: (i) subbasin-scale targeting of nutrient and sediment controls where winter–spring export pulses are projected to be largest; (ii) protection and restoration of riparian buffers and floodplain wetlands that can attenuate high-flow nutrient and sediment loads; and (iii) monitoring and management of stoichiometric changes, not just total loads, in ecologically sensitive reaches.

Finally, the modeled reductions in dissolved oxygen and changes in nutrient timing highlight risks for cold-water fisheries and other flow-sensitive biota. Earlier snowmelt and reduced summer baseflows can shrink cold-water habitat, while warmer temperatures and altered N:P ratios may favor species with different thermal and nutrient preferences (Marshall & Randhir, 2008; Grimm et al., 2013a; Grimm et al., 2013b). Management responses could include conserving groundwater-fed refugia, removing or modifying barriers that exacerbate thermal and hydrologic stresses, and integrating ecological flow criteria explicitly into climate adaptation planning. Overall, linking hydrologic regime shifts to nutrient dynamics and ecological risk provides a coherent basis for basin-scale climate adaptation in the Connecticut River watershed and illustrates how integrated modeling can inform strategic, climate-resilient water management.

### Limitations of the study

First, the analysis relies on the SWAT–HAWQS modeling framework driven by selected CMIP5 GCMs and RCP scenarios, introducing structural, parameter, and climate-projection uncertainties that may influence the magnitude and timing of simulated hydrologic and nutrient responses. Second, the study does not explicitly incorporate future land-use change, management adaptations, or point- and nonpoint-source policy interventions, which could substantially alter nutrient-loading trajectories under climate change. Third, while model performance metrics were within acceptable ranges, limitations in long-term water-quality monitoring data and the absence of sub-daily process representation constrain the ability to capture extreme-event dynamics and short-term nutrient pulses fully.

## Conclusion

Predictions of increased frequency and intensity of extreme weather events (IPCC, 2022; Burford et al., 2020) suggest these events will impact hydrological processes within the watershed. This study shows that these processes will change under the climate scenarios RCP 4.5 and RCP 8.5, with effects observable at both annual and monthly scales. Our results also reveal a shift in precipitation seasonality, with more rain expected in winter and early spring, which will cascade into other aspects of hydrological processes. Uncertainty is an important aspect to be assessed in watershed modeling (Tsvetkova & Randhir, 2019). The propagation of uncertainty from climatic to hydrologic variables is an important factor at daily and sub-daily scales in the watershed. Alternative stochastic methods could reveal more information on the climatic uncertainty, which is beyond the scope of this study. Stochastic methods that could be used in future work include stochastic rainfall generators (Blenkinsop et al., 2013; Burton et al., 2010; De Luca, Petroselli & Galasso, 2020; De Luca & Petroselli, 2021; Forsythe et al., 2014).

The observed changes in these processes will influence nutrient loading in the watershed. Several studies on the Connecticut River report similar findings (Marshall & Randhir, 2008; Paulding & Randhir, 2021). For instance, our results indicate shifts in nutrient seasonality, with peak phosphorus moving from August in the baseline to April under both climate scenarios. Peak nitrogen occurred in April under RCP 4.5 but remained in August under RCP 8.5. Increased evapotranspiration was also observed in both climate scenarios. Changes in evapotranspiration can affect soil moisture, impacting nutrient cycling (Howarth et al., 2006; USGCRP, 2017). A strong correlation exists between phosphorus and phytoplankton in freshwater, and between nitrogen and phytoplankton in marine and estuarine environments (Anderson, Gilbert & Burkholder, 2002). Both temperature and nutrient levels are essential, but the nitrogen-to-phosphorus ratio is also crucial (Anderson, Gilbert & Burkholder, 2002). This study finds that the nitrogen-to-phosphorus ratio decreased in every subwatershed and across the entire system under the climate change scenarios. However, previous research on the Connecticut River shows that the main stem is phosphorus-limited, while tributary subbasins are nitrogen-limited (Marshall & Randhir, 2008). Managing phosphorus and nitrogen together is essential for the Connecticut River as it flows into Long Island Sound.

Therefore, nitrogen must be managed within the freshwater system to prevent its downstream transport to marine waters, where it can cause eutrophication and harmful algal blooms. Studies indicate that when only phosphorus is managed, algal blooms can still occur downstream in marine waters; without freshwater algal growth, a significant portion of nitrogen would likely migrate (Burkholder & Glibert, 2013). Nutrient management at point sources, such as wastewater treatment plants, is regulated under the National Pollutant Discharge Elimination System (NPDES) permit program. Dual management of phosphorus and nitrogen is vital in the Connecticut River, as it drains into Long Island Sound. Therefore, nitrogen needs to be managed in the freshwater system to prevent its transport downstream to marine waters, where it may cause eutrophication and harmful algal blooms. Studies have shown that when only phosphorus was managed, a resultant algal bloom occurred downstream in marine waters; the absence of freshwater algal growth would have moved a large portion of the nitrogen (Burkholder & Glibert, 2013).

Managing nutrients at point sources such as wastewater treatment plants is regulated under the NPDES permit program. Continued upgrades of wastewater treatment facilities to the latest technology are recommended whenever possible. If all the treatment plants on the Connecticut River watershed upstream of Connecticut were able to reduce their nitrogen discharge to 3 mg/l, then about 33% of the nitrogen discharges to Long Island Sound from the Connecticut River would be eliminated (Evans, 2008).

Ongoing upgrades to wastewater treatment facilities, using the latest technologies, are recommended whenever possible. If all the treatment plants in the Connecticut River watershed upstream of Connecticut could reduce their nitrogen discharge to 3 mg/l, it would reduce about 33% of the nitrogen entering Long Island Sound from the Connecticut River (Evans et al., 2008). Combined Sewer Overflows (CSOs) pose a major problem in urban areas. During heavy rains, they release untreated sewage into rivers and tributaries. Retrofitting these systems is costly and slow; however, prioritizing infrastructure upgrades can help reduce peak nutrient discharges. Nonpoint sources of nutrient pollution are more difficult to control because of their widespread nature. Stormwater Best Management Practices (BMPs) should be developed to manage nutrients during peak flow periods. With increasing storm intensity and shifting timing caused by climate change, it is crucial that these systems can handle large volumes of water. Stormwater BMPs fall into two categories: structural and non-structural. Structural BMPs include bioretention ponds (rain gardens), swales, and permeable pavers. When designing these, nutrient treatment should be prioritized, as not all BMPs effectively control nutrient levels (O’Shea, Borst & Nietch, 2002). Non-structural BMPs involve education and source control, both of which are essential for successful implementation (Yang & Lusk, 2018). Agricultural BMPs, such as cover crops and riparian buffers, could reduce nitrogen loads to Long Island Sound by 34% if adopted across the entire watershed. Other measures, including fertilizer management, conservation tillage, crop rotation, and soil and fertilizer nutrient testing, can also decrease nutrient inflows into the Connecticut River and its tributaries (Sharpley et al., 2006).

## Additional Information and Declarations

### Data Availability

The following information was supplied regarding data availability: All datasets are available in the public domain at: https://hawqs.tamu.edu/#/help. The full details are available at https://hawqs.tamu.edu. The online software that has preloaded datasets to run the model is available at: HAWQS 2.0, 2023, “HAWQS System 2.0 and Data to model the lower 48 conterminous U.S using the SWAT model”, https://doi.org/10.18738/T8/GDOPBA, Texas Data Repository, V2.

## References

[ref-1] Allen RG (1986). A Penman for all seasons. Journal of Irrigation and Drainage Engineering.

[ref-2] Allen RG, Jensen ME, Wright JL, Burman RD (1989). Operational estimates of evapotranspiration. Agronomy Journal.

[ref-3] Anderson DM, Gilbert PM, Burkholder JM (2002). Harmful algal blooms and eutrophication: nutrient sources, composition, and consequences. Estuaries.

[ref-4] Arnold JG, Moriasi DN, Gassman PW, Abbaspour KC, White MJ, Srinivasan R, Santhi C, Harmel RD, Van Griensven A, Van Liew MW, Kannan N (2012). SWAT: model use, calibration, and validation. Transactions of the ASABE.

[ref-5] Bagnold RA (1966). An approach to the sediment transport problem from general physics. Prof. Paper 422-1.

[ref-6] Baker J, Randhir T (2022). Climate change impacts on nitrogen and phosphorus loading in New England watersheds. Journal of Nature and Spatial Sciences (JONASS).

[ref-7] Beskenis J (2006). Connecticut river watershed 2003 chlorophyll and periphyton technical memorandum. Connecticut river watershed 2003–2007 water quality assessment report E1 Appendix E.

[ref-8] Blenkinsop S, Harpham C, Burton A, Goderniaux P, Brouyère S, Fowler HJ (2013). Downscaling transient climate change with a stochastic weather generator for the Geer catchment, Belgium. Climate Research.

[ref-9] Brooks PD, Grogan P, Templer PH, Groffman P, Öquist MG, Schimel J (2011). Carbon and nitrogen cycling in snow-covered environments. Geography Compass.

[ref-10] Bruland GL, Richardson CJ (2006). An assessment of the phosphorus retention capacity of wetlands in the Painter Creek Watershed, Minnesota, USA. Water, Air, & Soil Pollution.

[ref-11] Burford MA, Carey CC, Hamilton DP, Huisman J, Paerl HW, Wood SA, Wulff A (2020). Perspective: advancing the research agenda for improving understanding of cyanobacteria in a future of global change. Climate Change and Harmful Algal Blooms.

[ref-12] Burkholder JM, Glasgow HB (1997). Pfiesteria piscicida and other Pfiesteria-like dinoflagellates: behavior, impacts, and environmental controls. Limnology and Oceanography.

[ref-13] Burkholder JM, Glasgow HB, Deamer-Melia NJ, Springer J, Parrow MW, Zheng C, Cancellieri P (2001). Species of the toxic Pfiesteria complex, and the importance of functional type in data interpretations. Environmental Health Perspectives.

[ref-14] Burkholder JM, Glibert PM, Levin SA (2013). Eutrophication and oligotrophication. Encyclopedia of biodiversity.

[ref-15] Burton A, Fowler HJ, Blenkinsop S, Kilsby CG (2010). Downscaling transient climate change using a Neyman–Scott rectangular pulses stochastic rainfall model. Journal of Hydrology.

[ref-16] Connecticut Department of Energy and Environmental Protection (CT DEEP) (2020). Cyanobacteria blooms in Connecticut.

[ref-17] De Luca DL, Petroselli A (2021). STORAGE (STOchastic RAinfall GEnerator): a user-friendly software for generating long and high-resolution rainfall time series. Hydrology.

[ref-18] De Luca DL, Petroselli A, Galasso L (2020). A transient stochastic rainfall generator for climate changes analysis at hydrological scales in Central Italy. Atmosphere.

[ref-19] Dodds WK (2007). Trophic state, eutrophication and nutrient criteria in streams. Trends in Ecology & Evolution.

[ref-20] Edwards W, Owens L (1991). Large storm effects on total soil erosion. Journal of Soil and Water Conservation.

[ref-21] Evans BM (2008). An evaluation of potential Nitrogen load reductions to long island sound from the Connecticut River basin. New England Interstate Water Pollution Control Commission.

[ref-22] Fant C, Srinivasan R, Boehlert B, Rennels L, Chapra SC, Strzepek KM, Corona J, Allen A, Martinich J (2017). Climate change impacts on U.S. water quality using two models: HAWQS and U.S. basins. Water.

[ref-23] Forsythe N, Fowler HJ, Blenkinsop S, Burton A, Kilsby CG, Archer DR, Harpham C, Hashmi MZ (2014). Application of a stochastic weather generator to assess climate change impacts in a semi-arid climate: the Upper Indus Basin. Journal of Hydrology.

[ref-24] Gassman PW, Reyes MR, Green CH, Arnold JG (2007). The soil and water assessment tool: historical development, applications, and future research directions. Transactions of the ASABE.

[ref-25] Gent PR, Danabasoglu G, Donner LJ, Holland MM, Hunke E, Jayne S, Lawrence D, Neale RB, Rasch PJ, Vertenstein M, Worley PH (2011). The community climate system model version 4. Journal of Climate.

[ref-26] Grimm NB, Chapin III FS, Bierwagen B, Gonzalez P, Groffman PM, Luo Y, Melton F, Nadelhoffer K, Pairis A, Raymond PA, Schimel J (2013a). The impacts of climate change on ecosystem structure and function. Frontiers in Ecology and the Environment.

[ref-27] Grimm NB, Chapin III FS, Bierwagen B, Gonzalez P, Groffman PM, Luo Y, Melton F, Nadelhoffer K, Pairis A, Raymond PA, Schimel J, Williamson CE (2013b). The impacts of climate change on ecosystem structure and function. Frontiers in Ecology and the Environment.

[ref-28] HAWQS 2.0 (2023).

[ref-29] HAWQS (2020). HAWQS system and data to model the lower 48 conterminous U.S using the SWAT model. Texas Data Repository.

[ref-30] Haygarth PM, Jarvis SC (2000). Phosphorus transfer from agricultural soils. Advances in Agronomy.

[ref-31] Hooghoudt SB (1940). Bijdragen tot de kennis van eenige natuurkundige grootheden van den grond: Algemeene beschouwing van het probleem van de detailontwatering en de infiltratie door middel van parallel loopende drains, greppels, slooten en kanalen. https://edepot.wur.nl/250838.

[ref-32] Howarth R, Chan F, Conley DJ, Garnier J, Doney SC, Marino R, Billen G (2011). Coupled biogeochemical cycles: eutrophication and hypoxia in temperate estuaries and coastal marine ecosystems. Frontiers in Ecology and the Environment.

[ref-33] Howarth RW, Swaney DP, Boyer EW, Marino R, Jaworski N, Goodale C, Martinelli LA, Howarth RW (2006). The influence of climate on average nitrogen export from large watersheds in the Northeastern United States. Nitrogen cycling in the Americas: natural and anthropogenic influences and controls.

[ref-34] IPCC (2022). Summary for Policymakers [H.-O. Pörtner, D.C. Roberts, E.S. Poloczanska, K. Mintenbeck, M. Tignor, A. Alegría, M. Craig, S. Langsdorf, S. Löschke, V. Möller, A. Okem (eds.)]. Climate Change 2022: Impacts, Adaptation, and Vulnerability. Contribution of Working Group II to the Sixth Assessment Report of the Intergovernmental Panel on Climate Change [H.-O. Pörtner, D.C. Roberts, M. Tignor, E.S. Poloczanska, K. Mintenbeck, A. Alegría, M. Craig, S. Langsdorf, S. Löschke, V. Möller, A. Okem, B. Rama (eds.)].

[ref-35] Jochem G (2019). Cyanobacteria algae bloom found in Northampton ponds. https://www.gazettenet.com/Toxic-Algae-Bloom-in-Northampton-28002176.

[ref-36] Marshall E, Randhir T (2008). Effect of climate change on watershed system: a regional analysis. Climatic Change.

[ref-37] Mass.Gov (2025). Harmful Cyanobacterial Bloom Advisories in Massachusetts. Mass.gov portal of the Commonwealth of Massachusetts.

[ref-38] McElroy AD, Chiu SY, Nebgen JW, Aleti A, Bennett FW (1976). Loading functions for assessment of water pollution from nonpoint sources. Environmental protection technical service, EPA 600/2-76-151.

[ref-39] Monteith JL (1965). Evaporation and the environment.

[ref-40] Moriasi DN, Arnold JG, Van Liew MW, Bingner RL, Harmel RD, Veith TL (2007). Model evaluation guidelines for systematic quantification of accuracy in watershed simulations. Transactions of the ASABE.

[ref-41] Neale RB, Richter J, Park S, Lauritzen PH, Vavrus SJ, Rasch P, Zhang M (2013). The mean climate of the community Atmosphere Model (CAM4) in forced SST and fully coupled experiments. Journal of Climate.

[ref-42] Neitsch SL, Arnold JG, Kiniry JR, Williams JR, King KW (2002). Soil and water assessment tool theory document.

[ref-43] O’Shea M, Borst M, Nietch C, Stecker EW, Huber WC (2002). The role of stormwater BMPS in mitigating the effects of nutrient over enrichment in the urban watershed.

[ref-44] Paerl HW, Huisman J (2009). Climate change: a catalyst for global expansion of harmful cyanobacterial blooms. Environmental Microbiology Reports.

[ref-45] Paulding C, Randhir TO (2021). An ecohydrological assessment of potential impacts of climate change on the herpetofauna. Sustainability and Climate Change.

[ref-46] Rallison RE, Miller N, Singh VP (1981). Past, present and future SCS runoff procedure. Rainfall runoff relationship.

[ref-47] Resilient MA Climate Change Clearinghouse for the Commonwealth (resilient MA) (2018). Massachusetts climate change projections. https://eea-nescaum-dataservices-assets-prd.s3.us-east-1.amazonaws.com/resources/production/MA%20Statewide%20and%20MajorBasins%20Climate%20Projections_Guidebook%20Supplement_March2018.pdf.

[ref-48] Ross ER, Randhir TO (2022). Effects of climate and land use changes on water quantity and quality of coastal watersheds of Narragansett Bay. Science of the Total Environment.

[ref-49] Sharpley AN, Daniel T, Gibson G, Bundy L, Cabrera M, Sims T, Stevens R, Lemunyon J, Kleinman P, Parry R (2006). Best management practices to minimize agricultural phosphorus impacts on water quality. ARS-163. USDA-ARS, Washington, DC. 2006 Jul 20.

[ref-50] Sharpley A, Jarvie HP, Buda A, May L, Spears B, Kleinman P (2013). Phosphorus legacy: overcoming the effects of past management practices to mitigate future water quality impairment. Journal of Environmental Quality.

[ref-51] Sharpley AN, Syers JK (1979). Phosphorus inputs into a stream draining an agricultural watershed: II. Amounts and relative significance of runoff types. Water, Air and Soil Pollution.

[ref-52] Soil Conservation Service (1972). Section 4: hydrology in national engineering handbook.

[ref-53] Stone JR, Stone BD (2012). Varve records from glacial lakes in southern New England; constraints on the timing of late-Wisconsinan deglaciation. Abstracts with Programs—Geological Society of America.

[ref-54] Talib A, Randhir TO (2017). Climate change and land use impacts on hydrologic processes of watershed systems. Journal of Water and Climate Change.

[ref-55] Tsvetkova O, Randhir TO (2019). Spatial and temporal uncertainty in climatic impacts on watershed systems. Science of the Total Environment.

[ref-56] US Environmental Protection Agency (2017). National water quality inventory: report to congress. EPA 841-R-16-011.

[ref-57] US Geological Survey (USGS) (2025). Climate change and future water availability in the United States. Professional Paper 1894-E.

[ref-58] USGCRP (2017). Climate science special report.

[ref-59] Williams JR (1975). Universal equation using runoff. ARS-S.

[ref-60] Williams JR, Hann RW (1978). Optimal operation of large agricultural watersheds with water quality constraints. Tech. Rept. No. 96.

[ref-61] Woodward G, Gessner MO, Giller PS, Gulis V, Hladyz S, Lecerf A, Malmqvist B, McKie BG, Tiegs SD, Cariss H, Dobson M, Elosegi A, Ferreira V, Graça MAS, Fleituch T, Lacoursière JO, Nistorescu M, Pozo J, Risnoveanu G, Schindler M, Vadineanu A, Vought LBM, Chauvet E (2012). Continental-scale effects of nutrient pollution on stream ecosystem functioning. Science.

[ref-62] Yang YY, Lusk M (2018). Nutrients in urban stormwater runoff: current state of the science and potential mitigation options. Current Pollution Reports.

[ref-63] Yen H, Daggupati P, White MJ, Srinivasan R, Gossel A, Wells D, Arnold JG (2016). Application of large-scale, multi-resolution watershed modeling framework using the hydrologic and water quality system (HAWQS). Water.

[ref-64] Zhao S, He W, Dong T, Zhou J, Xie X, Mei Y, Wan S, Jiang Y (2021). Evaluation of the performance of CMIP5 models to simulate land surface air temperature based on long-range correlation. Frontiers in Environmental Science.

